# Adverse effects of falciparum and vivax malaria and the safety of antimalarial treatment in early pregnancy: a population-based study

**DOI:** 10.1016/S1473-3099(11)70339-5

**Published:** 2012-05

**Authors:** R McGready, SJ Lee, J Wiladphaingern, EA Ashley, MJ Rijken, M Boel, JA Simpson, MK Paw, M Pimanpanarak, Oh Mu, P Singhasivanon, NJ White, FH Nosten

**Affiliations:** a Shoklo Malaria Research Unit, Mae Sot, Tak, Thailand; b Mahidol-Oxford Tropical Medicine Research Unit, Bangkok, Thailand; c Centre for Tropical Medicine, University of Oxford, Oxford, UK; d Centre for Molecular, Environmental, Genetic and Analytic Epidemiology, The University of Melbourne, Melbourne, Australia

## Abstract

**Background:**

The effects of malaria and its treatment in the first trimester of pregnancy remain an area of concern. We aimed to assess the outcome of malaria-exposed and malaria-unexposed first-trimester pregnancies of women from the Thai–Burmese border and compare outcomes after chloroquine-based, quinine-based, or artemisinin-based treatments.

**Methods:**

We analysed all antenatal records of women in the first trimester of pregnancy attending Shoklo Malaria Research Unit antenatal clinics from May 12, 1986, to Oct 31, 2010. Women without malaria in pregnancy were compared with those who had a single episode of malaria in the first trimester. The association between malaria and miscarriage was estimated using multivariable logistic regression.

**Findings:**

Of 48 426 pregnant women, 17 613 (36%) met the inclusion criteria: 16 668 (95%) had no malaria during the pregnancy and 945 (5%) had a single episode in the first trimester. The odds of miscarriage increased in women with asymptomatic malaria (adjusted odds ratio 2·70, 95% CI 2·04–3·59) and symptomatic malaria (3·99, 3·10–5·13), and were similar for *Plasmodium falciparum* and *Plasmodium vivax*. Other risk factors for miscarriage included smoking, maternal age, previous miscarriage, and non-malaria febrile illness. In women with malaria, additional risk factors for miscarriage included severe or hyperparasitaemic malaria (adjusted odds ratio 3·63, 95% CI 1·15–11·46) and parasitaemia (1·49, 1·25–1·78 for each ten-fold increase in parasitaemia). Higher gestational age at the time of infection was protective (adjusted odds ratio 0·86, 95% CI 0·81–0·91). The risk of miscarriage was similar for women treated with chloroquine (92 [26%] of 354), quinine (95 [27%) of 355), or artesunate (20 [31%] of 64; p=0·71). Adverse effects related to antimalarial treatment were not observed.

**Interpretation:**

A single episode of falciparum or vivax malaria in the first trimester of pregnancy can cause miscarriage. No additional toxic effects associated with artesunate treatment occurred in early pregnancy. Prospective studies should now be done to assess the safety and efficacy of artemisinin combination treatments in early pregnancy.

**Funding:**

Wellcome Trust and Bill & Melinda Gates Foundation.

## Introduction

Published evidence on the effects of malaria and antimalarial drug exposure during the first trimester of pregnancy is scarce.^1^, ^2^ This absence of data is because antenatal clinic services are usually unavailable in the rural tropics, and if they are available, women rarely present before the second trimester. Furthermore, estimation of gestational age is often imprecise, and pregnant women are usually excluded from drug studies.^1^, ^3^ Artemisinin combination treatments are recommended by WHO for the treatment of all *Plasmodium falciparum* malaria^4^ except in the first trimester because results from animal studies suggest that artemisinins are embryotoxic. These drugs are selectively toxic in a dose-dependent and time-dependent manner to primitive fetal erythroblasts.^5^ Experiments done on animals suggest that exposure during a limited time window in the first trimester (corresponding to 4–10 weeks of gestation or 6–12 weeks after menstruation in human beings^6^) results in fetal resorption or fetal loss.^6^, ^7^, ^8^, ^9^ Limb developmental abnormalities have been reported in rodents, but not in primates.^5^ In pregnant cynomolgus monkeys^6^ no effect on development (embryolethality or malformation) was reported, with treatment at 4 mg/kg per day of artesunate on gestational days 20 to 50 (ie, the “no observed adverse effect level” was 4 mg/kg per day). Early red-blood-cell formation in human beings is spread over many days compared with synchronous production over 1 day in rodents and hence the margin of safety with short courses of artemisinin treatment could be greater than initially thought.^9^

Pregnant women are at higher risk of severe malaria than are non-pregnant women of the same age.^10^ Both *P falciparum* and *Plasmodium vivax* malaria reduce birthweight in this setting.^11^, ^12^ No reliable measures for the prevention of malaria exist^13^, ^14^, ^15^ for pregnant women and their treatment options are limited by drug resistance.^16^

On the Thai–Burmese border the rapid spread of multidrug resistant *P falciparum* in the early 1990s^17^ resulted in the first large-scale use of an artemisinin combination treatment (mefloquine plus artesunate) for the treatment of uncomplicated falciparum malaria. Artesunate also proved safer and more effective than intravenous quinine for the treatment of uncomplicated hyperparasitaemia (>4% infected red blood cells)^18^ and severe malaria.^10^ Once a drug is used inadvertent exposures during pregnancy are inevitable. We aimed to assess the outcome of pregnancy in women who had a single episode of malaria in the first trimester (and no further episode later in pregnancy) compared with women in the first trimester of pregnancy who had no malaria. Additionally, we assessed the outcome of pregnancy after treatment with chloroquine-based, quinine-based, or artesunate-based antimalarials, for first trimester infection.

## Methods

### Study area and population

The Shoklo Malaria Research Unit (SMRU) is situated on the northwestern border of Thailand. Malaria transmission is low and seasonal in this region. Pregnant women have been encouraged to attend SMRU clinics providing antenatal care for the early detection and treatment of malaria since 1986 for refugees and since 1998 for migrants. Ethical approval for this audit of hospital records was given by the Oxford Tropical Research Ethics Committee (OXTREC 28-09). Data on first-trimester malaria from some of the same records have been published previously.^12^, ^19^, ^20^, ^21^

### Procedures

Women were encouraged to attend as soon as they were aware of their pregnancy. More than 90% of pregnant women in the camps for displaced persons where SMRU operates (current population 55 000) have attended antenatal clinics for regular malaria screening.^12^ At the first consultation, an obstetric and medical history was recorded, and a detailed clinical examination was done. History of malaria during pregnancy and information on date, place, malaria species, parasitaemia, and drug treatment was recorded. Information on smoking was gathered routinely from July, 1997. History of miscarriage was divided into none, a single previous miscarriage, and two or more miscarriages.^22^ At each weekly visit a finger-prick blood sample was examined for malaria parasites by trained microscopists. Haematocrit was measured every 2 weeks. Women were also encouraged to attend the antenatal clinic at any time if they felt unwell. Axillary or aural temperatures were recorded routinely. Although detailed diagnostics were available for some women with fevers (≥37·5°C) caused by diseases other than malaria,^23^ the diagnosis of non-malaria febrile illness relied mainly on clinical findings. Rates of HIV and syphilis were very low in the study population^24^ and have not been included in this analysis.

Severity of malaria was categorised into three groups: uncomplicated malaria; hyperparasitaemic malaria (parasitaemia ≥4% without signs of severity or evidence of vital-organ dysfunction); and severe malaria (signs of severity or severe organ dysfunction). Symptomatic malaria was defined as parasitaemia and a history of fever in the past 48 h or a measured axillary or aural temperature of 37·5°C or more.^25^ Asymptomatic malaria was defined as slide-confirmed malaria with no history of fever in the previous 48 h and a temperature of less than 37·5°C. No presumptive treatment of malaria was given. Severe anaemia was defined as a haematocrit less than 20%.

Patients with vivax malaria were treated with oral chloroquine 10 mg base per kg on day 0, 10 mg base per kg on day 1, and 5 mg base per kg on day 2. Patients with falciparum malaria were treated with oral quinine 10 mg salt per kg three times a day for 7 days or artesunate (outside the first trimester), usually 2 mg/kg per day for 7 days (total dose 10–16 mg/kg). Since 2007, clindamycin (5 mg/kg three times a day for 7 days) was added to quinine as first-line treatment in the first trimester. Artesunate was prescribed in the first trimester for quinine treatment failures, uncomplicated hyperparasitaemia, or severe malaria,^4^ although before 1993 these patients were treated with intravenous quinine. Women also received thiamine (vitamin B_1_, 100 mg a day) to prevent infant mortality from beri-beri, and ferrous sulphate and folic acid at prophylactic doses (200 mg a day and 5 mg a week, respectively) or treatment doses (200 mg three times a day and 5 mg a day, respectively). Delivery with the assistance of trained midwives in the SMRU delivery unit was encouraged, but home birth with a traditional birth attendant was still practised. All newborns, including those born at home, were examined by trained midwives and were weighed with Salter scales (Salter, Birmingham, UK; accurate to 50 g).^26^

First trimester was defined as an estimated gestational age less than 14 weeks. Methods to estimate gestational age included ultrasound,^27^ the Dubowitz newborn assessment,^28^ a fundal height formula validated for this population,^29^ or last menstrual period. Ultrasound at 7–13 weeks can measure gestational age to within 3 days, but scans done later, 14–22 weeks, decline in accuracy and can measure gestational age to within 7 days.^27^ The Dubowitz examination in term infants can measure gestational age to within 14 days of the examination,^28^ and the fundal height formula varies in accuracy from −16 to 14 days.^29^ All infants had a surface examination at delivery by midwives using a standardised newborn head-to-toe examination, with cardiovascular examination routinely done since 2006 and all major abnormalities recorded and verified by a physician.

We reviewed all records of pregnant women attending the antenatal clinic until delivery from May 12, 1986, to Oct 31, 2010. The primary objective of the investigation was to assess the effects of malaria on pregnancy outcomes in the first trimester, and to compare the effects of different antimalarial drugs. To avoid the confounding effects of multiple malaria episodes and exposure to different antimalarial drugs during pregnancy, we selected women who began attending the antenatal clinic in their first trimester and if they had malaria (ie, only those with a single first-trimester episode). Women with no valid estimate of gestation, who had malaria in the second or third trimesters, or who had an unknown outcome were excluded from this analysis.

Analysis of birthweight was restricted to liveborn, congenitally normal, singleton infants weighed in the first 72 h of life. Miscarriage was defined as a pregnancy ending before 28 weeks, estimated gestational age. Termination of pregnancy was not available in this remote area. Stillbirth was a delivery from 28 weeks or an infant with a birthweight of 800 g or more in which the infant displayed no sign of life (gasping, muscular activity, cardiac activity). The 28-week estimated gestational age, rather than the current WHO 22-week cut-off for estimated gestational age was chosen, because no infant respiratory support was available in the clinics. Preterm births were deliveries before 37 weeks estimated gestational age. A non-viable conceptus was defined either by ultrasound examination (absent fetal heart beat, anembryonic gestation, retained products of conception, ectopic pregnancy, hydatidiform mole) or clinically (when ultrasound was not available) by significant vaginal bleeding in early pregnancy, open cervix on examination, passage of products of conception, or a negative pregnancy test. The date of expulsion of the uterine contents either spontaneously or by the use of medical or surgical intervention was taken as the date of miscarriage. The interval from treatment to date of miscarriage was not used in primary analysis because the capacity to diagnose non-viability and remove the uterine contents changed over time. Some women discontinued antenatal care before the outcome of pregnancy was known, usually because they left the study area.

### Statistical analysis

Data were analysed with SPSS (version 14·0) and STATA (version 11). Comparisons were made using the Student's *t* test or Mann-Whitney U test. Categorical variables were compared using the χ^2^ test or Fisher's exact test. To assess the role of malaria as a risk factor for miscarriage, malaria (symptomatic or asymptomatic) versus no malaria, number of pregnancies (multiple pregnancies *vs* first pregnancy), smoking status (yes or no), age of mother (13–20, 21–25, 26–30, ≥31 years), number of miscarriages (none, one, or two or more), and non-malaria febrile illness in the first trimester were examined initially with univariable logistic regression analysis with miscarriage as the outcome variable. We used multivariable logistic regression analysis to estimate the odds for miscarriage associated with malaria after accounting for age, smoking, women in their first pregnancy, number of previous miscarriages, and non-malaria febrile illness. Analyses were also repeated with adjustment for year of enrolment and the results were unchanged (data not shown). Adjusted population attributable fractions of miscarriage due to symptomatic and asymptomatic malaria, and falciparum and vivax malaria, were calculated using the aflogit module for STATA.^30^

To distinguish the effects of *P falciparum* and *P vivax* on the risk of miscarriage, and to estimate the associations between malaria severity, baseline log parasitaemia, and the risk of miscarriage, we did a subgroup analysis of women with malaria during the first trimester and excluded women with mixed infections (ten), episodes of *Plasmodium malariae* or *Plasmodium ovale* (eight), or unknown species (19). Birth outcomes were also compared between women treated with chloroquine, quinine, and artesunate, and women who had no malaria during pregnancy. A one-way ANOVA was used to compare mean estimated gestational age at birth and mean birthweight across the groups, and the χ^2^ test was used to compare the proportion of stillbirths and congenital abnormalities.

### Role of the funding source

The sponsor of the study had no role in study design, data collection, data analysis, data interpretation, or writing of the report. The corresponding author had full access to all the data in the study and had final responsibility for the decision to submit for publication.

## Results

Of 17 613 eligible women 15 735 (89%) presented to the SMRU clinic for antenatal care because they had a normal pregnancy (figure); others attended the clinic with signs of pregnancy loss (1196 [7%]), symptomatic malaria (microscopy confirmed; 280 [2%]), symptoms of illness (not malaria; 398 [2%]), or both symptomatic malaria and symptoms of other illness (four [<1%]). The proportions of teenagers, women in their first pregnancies, smokers, and those with a history of previous miscarriage, were higher in women with malaria compared with those with no malaria (table 1). Smoking increased with age. Slight demographic differences existed between the 30 813 excluded and 17 613 included women; excluded women were slightly younger, with more teenagers and women in their first pregnancy (webappendix p 1). Women with unknown pregnancy outcomes (2376) were slightly younger, more likely to be in their first pregnancy, and to have malaria within the first trimester than were women with known pregnancy outcomes (17 613). The distribution of antimalarial treatments was similar in the two groups (webappendix p 1).

**Figure:**
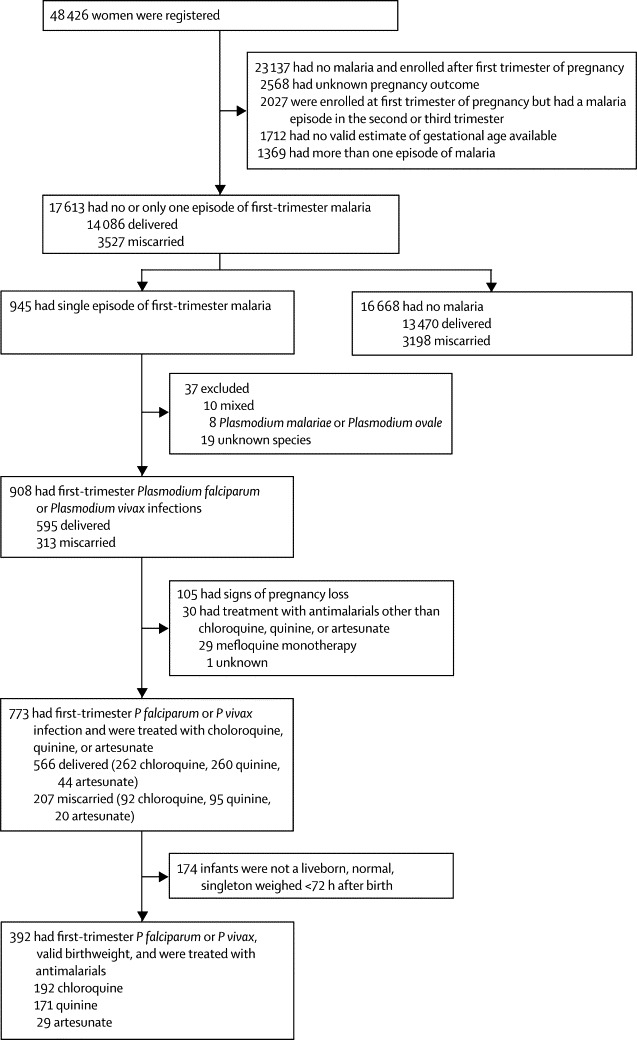
Study profile

**Table 1:** Demographic characteristics

		**No malaria in pregnancy (16 668)**	**First trimester malaria (945)**	**p value**
Age, years*		27 (7; 13–52)	26 (7; 13–45)	<0·0001
Teenagers, <20 years of age*		2416 (15%)	189 (20%)	<0·0001
Number of pregnancies†		3 (2–5; 1–17)	3 (1–5; 1–13)	0·0171
Parity‡		2 (1–3; 0–13)	1 (0–3; 0–10)	0·0002
Women in their first pregnancy†		3369 (20%)	257 (27%)	<0·0001
Smokers§		4095 (30%)	225 (37%)	0·0002
Previous miscarriage¶		4759 (29%)	315 (33%)	0·002
Smoking by age category				
	13–20 years	3587 (22%)	280 (30%)	<0·0001
	21–25 years	4154 (25%)	226 (24%)	0·5098
	26–30 years	4230 (25%)	199 (21%)	0·0029
	≥31 years	4696 (28%)	240 (25%)	0·0690
Non-malaria febrile illness*		293 (2%)	15 (2%)	0·7855
Antimalarial Treatment				
	Chloroquine	NA	429 (45%)	..
	Quinine	NA	390 (41%)	..
	Artemisinin derviative	NA	92 (10%)	..
	Mefloquine	NA	32 (3%)	..
	Other or unknown	NA	2 (<1%)	..
Median number of antenatal consultations for women with delivery		26 (13–29; 1–40)	24 (4–26; 1–37)	<0·0001
Miscarriage		3198 (19%)	329 (35%)	<0·0001

Data are mean (SD; range), median (IQR; range), or number (%). NA=not applicable.

* Data missing for one patient in no malaria group.

† Data missing for 27 patients in the no malaria group and one patient in malaria group.

‡ Data missing for 36 patients in the no malaria group and one patient in the malaria group.

§ Data missing for 2887 patients in the no malaria group and 336 patients in the malaria group.

¶ Data missing for 104 patients in the no malaria group and three patients in the malaria group.

The interval in days from the start of treatment of malaria to the date of the miscarriage was examined for each antimalarial treatment group (table 2). In pregnancies dated with the optimum technique, ultrasound, no significant difference was reported. The gestation at the start of diagnosis, equivalent to the date of treatment, was used for the following part of the analysis.

**Table 2:** Interval in days from treatment of malaria to date of miscarriage according to treatment group

	**Chloroquine**	**Quinine**	**Artesunate**
All women*	25 (0–169; 16–44; 92)	18 (1–172; 7–42; 95)	47 (0–91; 15–87; 20)
Ultrasound confirmed gestation†	25 (0–101; 14–42; 42)	19 (1–172; 7–42; 27)	37·5 (4–73; 10–51; 8)

Data are median (range; IQR; number of women).

* p value for chloroquine versus quinine=0·041; p value for chloroquine versus artesunate=0·061; p value for quinine versus artesunate=0·027.

† p value for chloroquine versus quinine=0·526; p value for chloroquine versus artesunate=0·450; p value for quinine versus artesunate=0·479.

Of 17 613 pregnant women, 14 086 (80%) delivered and 3527 (20%) miscarried. Severe anaemia in the first trimester in this cohort was infrequent (18 [<1%] of 16 483; 16 in women with malaria, and two in the non-malaria group), and it has not been included in further analysis. Significant risk factors for miscarriage included malaria (asymptomatic or symptomatic), having had more than one pregnancy, smoking, older maternal age, number of previous miscarriages, and non-malaria febrile illness in the first trimester (table 3). Since women in their first pregnancy cannot have had a previous miscarriage, we included number of previous miscarriages (and excluded the variable women who had had more than one pregnancy) in the multivariable logistic regression analyses. The results were unchanged if we adjusted for number of pregnancies instead of number of previous miscarriages. To ensure that exclusion of women without smoking data did not bias the results, we repeated the analyses with only the exposure variables: malaria, maternal age, number of miscarriages, and non-malaria febrile illness, in the logistic regression model. The results were unchanged. The highest odds for miscarriage were for women with symptomatic malaria compared with women with no malaria (table 3). Importantly, asymptomatic malaria was also associated with miscarriage. The population attributable fraction of miscarriage due to asymptomatic malaria was lower than symptomatic malaria, and for non-malaria febrile illness.

**Table 3:** Risk factors associated with miscarriage in women with either no malaria or a single malaria episode in the first trimester and known outcomes

	**Miscarriage (3527)**	**Delivery (14 086)**	**Unadjusted odds ratio**	Adjusted odds ratio*	**Population attributable fraction**
**Malaria symptoms**†					
No malaria	92% (3198)	97% (13470)	Reference group	Reference group	..
Asymptomatic malaria	3% (104)	2% (213)	2·06 (1·62–2·61; p<0·0001)	2·70 (2·04–3·59; p<0·0001)	1·4% (1·0–1·9)
Symptomatic malaria	5% (157)	1% (172)	3·84 (3·09–4·79; p<0·0001)	3·99 (3·10–5·13; p<0·0001)	2·6% (2·1–3·1)
**Pregnancy**					
Women in their first pregnancy	15% (537)	22% (3089)	Reference group	Reference group	..
Women who have had more than one pregnancy‡	85% (2986)	78% (10973)	1·57 (1·42–1·73; p<0·001)	..§	..
**Smoking**¶					
Non-smoker	62% (1817)	72% (8253)	Reference group	Reference group	..
Smoker	38% (1129)	28% (3191)	1·61 (1·48–1·75; p<0·0001)	1·26 (1·15–1·38; p<0·0001)	5·8% (3·4–8·1)
**Age**‖					
13–20 years	16% (558)	23% (3309)	Reference group	Reference group	..
21–25 years	19% (668)	26% (3712)	1·07 (0·94–1·21; p=0·295)	0·97 (0·84–1·11; p=0·627)	..
26–30 years	23% (825)	26% (3604)	1·36 (1·21–1·53; p<0·0001)	1·21 (1·06–1·39; p=0·005)	3·3% (1·0–5·5)
≥31 years	42% (1476)	25% (3460)	2·53 (2·27–2·82; p<0·0001)	2·16 (1·90–2·46; p<0·0001)	18·9% (15·9–21·8)
Number of previous miscarriages**					
None	61% (2133)	74% (10298)	Reference group	Reference group	..
One	24% (857)	19% (2671)	1·55 (1·42–1·69; p<0·0001)	1·31 (1·18–1·45; p<0·0001)	4·4% (2·7–6·1)
Two or more	15% (518)	7% (1029)	2·43 (2·17–2·73; p<0·0001)	1·89 (1·65–2·17; p<0·0001)	5·3% (4·0–6·5)
**Non-malaria febrile illness in the first trimester**					
No	96% (3402)	99% (13903)	Reference group	Reference group	..
Yes	4% (125)	1% (183)	2·79 (2·22–3·52; p<0·0001)	3·00 (2·34–3·84; p<0·0001)	2·1% (1·6–2·7)

Data are number (%), unadjusted or adjusted odds ratio (95% CI), or population attributable fraction (95% CI).

* n=14 190 (results unchanged if smoking variable excluded from multivariable logistic regression model [n=17 207]).

† Data missing for 68 patients in the miscarriage group and 231 patients in the delivery group.

‡ Data missing for four patients in the miscarriage group and 24 patients in the delivery group.

§ Highly collinear with number of previous miscarriages since women in their first pregnancy cannot have a previous miscarriage; estimated adjusted odds ratios for the other variables were unchanged whether parity or number of previous miscarriages were adjusted for or not.

¶ Data missing for 581 patients missing in the miscarriage group and 2642 in the delivery group.

‖ Data missing for one patient in the miscarriage group.

** Data missing for 19 patients in the miscarriage group and 88 patients in the delivery group.

Of 945 single episodes of malaria in the first trimester 496 were *P falciparum* only, 412 *P vivax* only, ten were mixed infections, eight *P malariae* or *P ovale* only, and 19 were not speciated (figure). Among the 908 women with *P falciparum* or *P vivax* monoinfections 595 (66%) women delivered and 313 (34%) miscarried. Maternal age, smoking history, number of previous miscarriages, estimated gestational age at the time of malaria episode, and parasitaemia were all significantly associated with miscarriage (515 women in the final model). The odds of miscarriage nearly doubled when maternal age was 31 years or older, and were twice as high among smokers (table 4). The odds of miscarriage increased 50% per ten-fold increase in parasitaemia. Only 24 women had uncomplicated hyperparasitaemia or severe malaria and this group had a higher odds of miscarriage than did those with uncomplicated malaria (adjusted odds ratio 3·63, 95% CI 1·15–11·46). The risks of miscarriage were similar in women with *P vivax* or *P falciparum* (table 4). Analysis of data from all women and adjustment for maternal age, previous miscarriages, smoking, and non-malaria febrile illness showed that the odds of miscarriage were about three times higher for women infected with *P falciparum* (adjusted odds ratio 2·7, 95% CI 2·1–3·4) or *P vivax* (3·1, 2·4–3·9) malaria than they were for women with no malaria. The population attributable fractions of miscarriage due to *P vivax* (2·2%, 95% CI 1·6–2·8) and *P falciparum* malaria (1·9%, 1·3–2·4) were similar. The proportion of miscarriage in pregnancies unaffected by malaria (19% [3198]) was similar to that seen in developed countries (10–20%), as are the associations with previous miscarriage, older maternal age, and smoking (table 3).^31^

**Table 4:** Risk factors associated with miscarriage in women with a single first-trimester episode of *Plasmodium vivax* or *Plasmodium falciparum*

		**Miscarriage (313)**	**Delivery (595)**	**Unadjusted odds ratio (95% CI; p value)**	Adjusted odds ratio (95% CI; p value)*
Malaria symptoms†					
	Asymptomatic	39% (100)	56% (211)	Reference group	Reference group
	Symptomatic	61% (154)	44% (169)	1·92 (1·39–2·66; p=0·0001)	1·42 (0·95–2·14; p=0·089)
Severity of malaria‡					
	Uncomplicated	95% (297)	98% (580)	Reference group	Reference group
	Severe or hyperparasitaemic	5% (14)	2% (10)	2·73 (1·20–6·23; p=0·017)	3·63 (1·15–11·46; p=0·028)
Species					
	*Plasmodium falciparum*	52% (163)	56% (333)	Reference group	Not included
	*Plasmodium vivax*	48% (150)	44% (262)	1·17 (0·89–1·54; p=0·263)	Not included
Pregnancy					
	Women in their first pregnancy	25% (78)	28% (169)	Reference group	Not included
	Women who have had more than one pregnancy§	75% (235)	72% (425)	1·20 (0·88–1·64; p=0·256)	Not included
Smoking¶					
	Non-smoker	51% (122)	71% (252)	Reference group	Reference group
	Smoker	49% (118)	29% (104)	2·34 (1·67–3·30; p<0·0001)	2·08 (1·43–3·02; p=0·0007)
Age					
	13–20 years	27% (84)	31% (187)	Reference group	Reference group
	21–25 years	17% (54)	28% (166)	0·72 (0·49–1·08; p=0·114)	0·78 (0·47–1·29; p=0·333)
	26–30 years	21% (66)	20% (120)	1·22 (0·82–1·82; p=0·316)	1·30 (0·78–2·15; p=0·318)
	≥31 years	35% (109)	21% (122)	1·99 (1·38–2·86; p=0·0002)	1·85 (1·16–2·96; p=0·010)
Number of previous miscarriages‖					
	None	62% (194)	68% (406)	Reference group	Not included
	One	25% (79)	21% (126)	1·31 (0·94–1·82; p=0·106)	Not included
	Two or more	12% (38)	10% (62)	1·28 (0·83–1·99; p=0·266)	Not included
Non-malaria febrile illness in the first trimester					
	No	97% (305)	99% (590)	Reference group	Not included
	Yes	3% (8)	1% (5)	3·10 (1·00–9·54; p=0·049)	
Calculated estimated gestational age at malaria episode, weeks (median [IQR])		8·0 (5·7–10·5)††	10·2 (7·6–12·3)††	0·88 (0·85–0·92; p<0·0001)	0·86 (0·81–0·91; p<0·0001)
Parasitaemia, μL (geometric mean [95% CI; number of women])**		1020 (726–1432; 262)‡‡	300 (238–378; 413)‡‡	1·52 (1·32–1·75; p<0·0001)§§	1·49 (1·25–1·78; p<0·0001)§§

Data are unadjusted or adjusted odds ratio (95% CI).

* The variables malaria (asymptomatic or symptomatic), severity of malaria, and parasitaemia were fitted in separate models because of collinearity, with each model adjusting for age, smoking, and calculated estimated gestational age at malaria episode.

† Data missing for 59 patients in the miscarriage group and 215 in the delivery group.

‡ Data missing for two patients in the miscarriage group and five in the delivery group.

§ Data missing for one patient in the delivery group.

¶ Data missing for 73 patients in the miscarriage group and 239 in the delivery group.

‖ Data missing for two patients in the miscarriage group and one in the delivery group.

** Data missing for 51 patients in the miscarriage group and 182 in the delivery group.

†† Data are median (IQR).

‡‡ Data are geometric mean (95% CI; number of women).

§§ Relative change in odds for miscarriage associated with a ten-fold increase in parasitaemia.

Single course treatments were with chloroquine (354), quinine (355), or artesunate (64). Intentional treatment with artemisinins was predominantly monotherapy (21 of 30 women) whereas inadvertent treatment (34 women) was all with an artemisinin combination treatment (table 5).

**Table 5:** Artemisinin treatments given to women with a single episode of malaria in the first trimester of pregnancy with a known pregnancy outcome

		**Delivery**	**Miscarriage**	**Total**
Intentional treatment		..	..	30
	Artesunate monotherapy	10 (59%)	7 (41%)	..
	Artesunate and clindamycin	6 (67%)	3 (33%)	..
	Artesunate (rescue after quinine)	2 (50%)	2 (50%)	..
Inadvertent treatment		..	..	34
	Artesunate and atovaquone-proguanil	1 (100%)	0	..
	Artesunate and doxycycline	1 (100%)	0	..
	Artemether-lumefantrine	1 (100%)	0	..
	Dihydroartemisinin and piperaquine	2 (100%)	0	..
	Artesunate and mefloquine	21 (72%)	8 (28%)	..
Total		44 (69%)	20 (31%)	64

Data are number (%)

Of 773 women treated for malaria, 566 (73%) delivered and 207 (27%) miscarried: 92 (26%) of 354 after treatment with chloroquine, 95 (27%) of 355 after treatment with quinine, and 20 (31%) of 64 after treatment with artesunate (p=0·71), despite the use of artesunate for high-risk groups (treatment failures and uncomplicated hyperparasitaemia or severe malaria). Among 34 women inadvertently given an artemisinin combination treatment, eight (24%) misscarried. This percentage was significantly lower than for miscarriage in women treated for uncomplicated hyperparasitaemia or severe malaria (nine [56%] of 16; p=0·030), but did not differ significantly when compared with miscarriage after intentional treatment of uncomplicated malaria (three [21%] of 14; p=0·072). Women with severe malaria or uncomplicated hyperparasitaemia usually received 16 mg/kg total dose of artesunate over 7 days whereas women with uncomplicated malaria received 12–14 mg/kg over 7 days.

Drug exposures between 6 and 12 weeks, gestation were investigated further: miscarriage proportions were 40% (ten of 25) for women treated with artesunate compared with 26% (51 of 193; p=0·162) for quinine and 30% (64 of 215; p=0·360) for chloroquine. After adjusting for the severity of *P falciparum* infection the risk for miscarriage in recipients of artesunate and quinine was not significantly different (adjusted odds ratio 1·22, 95% CI 0·45–3·27; p=0·70). Allowing for a plus or minus 14-day error on estimation of gestational age and so broadening the exposure window to between 4 weeks and less than 14 weeks, the miscarriage proportions were 34% (17 of 50) for women who received artesunate compared with 26% (83 of 323; p=0·232) for quinine and 26% (87 of 329; p=0·307) for chloroquine. Adverse effects related to treatment were not seen with quinine, chloroquine, or artesunate, although the numbers available for the artesunate group were small.

A single episode of malaria in the first trimester did not predispose to later stillbirth (table 6). The birthweight of liveborn infants in the non-malaria group (mean 2970 g [SD 451; range 900–5080]; n=11204) was not significantly higher than in the malaria group (2940 g [471; 780–4300]; n=392; p=0·217). Birthweights after first-trimester *P falciparum* (2962 g [SD 477; range 780–4300]; n=200) and *P vivax* infections (2917 g [465; 790–4130]; n=192; p=0·341) were similar. More infants born to mothers who had first-trimester malaria than those born to mothers without malaria were either never weighed or were weighed after 72 h (table 6). The overall proportion of congenital abnormalities did not differ significantly between groups (table 6, webappendix p 2).

**Table 6:** Neonatal outcomes of pregnancies followed up from the first trimester by antimalarial treatment group

		**No malaria, no treatment (13 470)**	**Chloroquine (262)**	**Quinine (260)**	**Artesunate (44)**	**p value**
Stillbirths*		153/13158 (1%)	3/254 (1%)	7/257 (3%)	1/44 (2%)	0·131
Male†		6718/13 315 (50%)	146/259 (56%)	144/253 (57%)	23/44 (52%)	0·055
Congenital abnormality‡		198/13 339 (1%)	1/260 (<1%)	2/258 (1%)	2/44 (4·5)	0·117
Twins		134/13 470 (1%)	0	1/260 (<1%)	1/44 (2%)	0·225
Estimated gestational age at birth, weeks		39·0 (1·8 [28·0–45·5])	38·8 (2·2 [28·0–44·0])	38·9 (2·1 [28·3–42·9])	38·9 (1·8 [32·6–41·4])	0·194
Birthweight not measured§		390/13 470 (3%)	13/262 (5%)	19/260 (7%)	4/44 (9%)	<0·0001
Liveborn normal singleton infants§						
	Birthweight measured <72 h	11 204/12 204 (89%)	192/239 (80%)	171/233 (73%)	29/38 (76%)	<0·0001
	Birthweight, g	2970 (451 [900–5080])	2917 (465 [790–4130])	2950 (488 [780–4300])	3035 (408 [2250–3900])	0·318

Data are number (%) or mean (SD [range]).

* Data missing for 312 patients in the no malaria, no treatment group, eight in the chloroquine group, and three in the quinine group.

† Data missing for 155 patients in the no malaria, no treatment group, three in the chloroquine group, and seven in the quinine group.

‡ Data missing for 131 patients in no malaria, no treatment group, two in the chloroquine group, and two in the quinine group.

§ Weight measured and completed within 72 h of birth.

## Discussion

This study is the largest to assess the effects of malaria and its treatment in the first trimester of pregnancy (panel). The study was undertaken in a remote rural area, in a community exposed to low seasonal transmission of *P falciparum* and *P vivax.* Despite the terrain, antenatal clinic attendance was very high. Malaria in pregnancy is a major cause of maternal mortality and low birthweight, which predisposes to neonatal mortality. Drug safety concerns still restrict the recommended treatment for falciparum malaria in the first trimester to a 7 day course of quinine—a poorly tolerated, poorly adhered to, and therefore ineffective treatment.

Panel **Research in context**

**Systematic review**

We searched PubMed for articles published up to August 24, 2011 using the search terms “malaria and miscarriage (or abortion)”. No prospective trials, enrolling women in early pregnancy that examine the risk associated with malaria infection were identified. We did another search using the search term “first trimester antimalarials (or quinine or chloroquine or artesunate)”. No published systematic reviews or randomised trials investigating treatment of malaria in the first trimester of pregnancy were identified. Attempts to ascertain efficacy and safety of antimalarial treatment in the first trimester in malaria-endemic countries are limited by small sample sizes and a failure to confirm malaria or gestational age at exposure. Studies assessing first-trimester antimalarial treatment of malaria are anecdotal, or at best observational.

**Interpretation**

Our results show that a single episode of symptomatic or asymptomatic *Plasmodium falciparum* or *Plasmodium vivax* malaria can cause miscarriage. Adverse effects related to treatment were not seen with quinine, chloroquine, or artesunate but the numbers available for the artesunate group are small. A randomised trial of first-trimester artemisinin-based treatment is now needed to make firm recommendations on the safety of first-trimester malaria treatments with this class of antimalarial drug.

Malaria in the first trimester was associated with miscarriage, and the association is likely to be causal. Risk of miscarriage was higher for women with symptomatic and asymptomatic malaria than it was for women who did not have malaria in the first trimester (table 3). The odds were similar with *P falciparum* and *P vivax.* Thus both vivax and falciparum malaria contribute significantly to avoidable fetal and infant death. This finding emphasises the importance of early detection of malaria and prompt effective treatment for all pregnant women. The comparably increased odds of miscarriage in non-malaria febrile illnesses suggest a pathogenic role of fever and proinflammatory responses,^32^ while the increased risk of abortion with asymptomatic malaria suggests a more malaria-specific pathological process.

A single successfully treated episode of malaria in the first trimester without later infection did not significantly affect birthweight. This suggests either a minimal effect of first-trimester infections on fetal growth or an efficient recovery during the remaining gestation. Later in pregnancy, the importance of malaria-specific pathogenic processes such as those related to placental cytoadherence presumably increases. Nevertheless, these findings have serious implications for malaria treatment and prevention policies, which currently ignore the first trimester. Early detection and treatment will reduce the effects of symptomatic malaria on miscarriage but only prevention can eliminate the increased risk of miscarriage in women with asymptomatic disease. Preventive options are limited, especially in terms of effective and safe prophylaxis. Symptomatic malaria infections are less common in areas of high transmission, where women have greater immunity than in areas of low transmission, but the increased risk of miscarriage with asymptomatic malaria is of major concern because the prevalence of asymptomatic malaria parasitaemia in such areas is very high. Prospective studies in high-transmission settings are needed to assess these risks.

Our analysis could not account for several factors: inherent errors in assessments of gestational age, pregnancy intervals, use of contraceptives (low uptake in this population), the fact that heavy bleeding can be reported as miscarriage, the possibility of confounding bacterial vaginosis, and environmental factors such as DDT (dichlorodiphenyltrichloroethane) exposure.^33^

Data on the safety of artemisinin derivatives are reassuring. We did not record high rates of fetal resorption, which have been reported in animal studies.^6^, ^7^, ^8^, ^9^ Globally, 333 first-trimester exposures to artemisinin derivatives have been reported with known pregnancy outcomes: Deen and colleagues^34^ reported 77 pregnancies with an unknown malaria status and Manyando and colleagues^35^ reported 106; Adam and colleagues^36^ reported 62 pregnancies with a confirmed malaria diagnosis and we have reported 44. No significant adverse effects have been recorded. Artemisinin derivatives were not used routinely in early pregnancy in our study sites. Miscarriage rates in the 24 first-trimester episodes of hyperparasitaemia or severe malaria were high but artesunate did not result in higher rates of miscarriage than did quinine. Antimalarials, especially artemisinin derivatives in this cohort, were not associated with miscarriage after controlling for maternal and malaria characteristics. Birth outcomes did not differ significantly after first-trimester antimalarial treatment with artesunate compared with the other antimalarials, quinine and chloroquine. No significant excess of malformations was reported. Overall, these results suggest that the adverse effects of malaria in the first trimester substantially outweigh any adverse effects of its treatment. Nevertheless, further information, preferably from a randomised trial of artemisinin combination treatment in the first trimester, is needed before definitive statements on safety can be made.

This study shows that, in the context of ready access to effective treatment, the adverse effects of falciparum and vivax malaria are similar and substantial in early pregnancy. Malaria control measures must be directed against all malarias (including asymptomatic infections). The time has come to reassess the treatment of malaria in early pregnancy.

## Web Extra Material

Supplementary webappendix
