# Supplementary material for: Adverse effects of falciparum and vivax malaria and the safety of antimalarial treatment in early pregnancy: a population-based study
Source: Lancet Infect Dis. 2012 May;12(5):388–96. doi: 10.1016/S1473-3099(11)70339-5 (PMC3346948; doi:10.1016/S1473-3099(11)70339-5)
Supplement: Supplementary webappendix [file mmc1.pdf]

THE LANCET Infectious Diseases

Supplementary webappendix

This webappendix formed part of the original submission and has been peer reviewed. We post it as supplied by the authors.

Supplement to: McGready R, Lee SJ , Wiladphaingern J, et al. Adverse effects of falciparum and vivax malaria and the safety of antimalarial treatment in early pregnancy: a population-based study. *Lancet Infect Dis* 2011; published online Dec 13. DOI:10.1016/S1473-3099(11)70339-5.

Supplementary Table:

Table 1

Characteristics of women included and excluded from the analysis (n=48,426)

Characteristic	Included N=17,613	Excluded N=30,813
Age, yrs, mean ± SD, [range]	27 ±7 (21-32) [13-52]	26±7 (20-30) [13-50]
Teenager < 20 y.o., n (%)	2605/17,612 (14.8)	4245/30,704 (17.1)
Gravidity, median (IQ range) [range]	3 (2-5) [1-17]	3 (1-4) [1-19]
Parity, median (IQ range) [range]	2 (1-3) [0-13]	2 (0-3) [0-17]
Primigravidae, n (%)	3,626/17,585 (20.6)	8,352/30,615 (27.3)
Smokers, n (%)	4320/14,390 (30.0)	6,435/20,512(31.4)

IQ range – inter-quartile range; SD - standard deviation.
Missing data: Age and teenager n=110, primigravidae n=226, parity n=226, gravidity n=244, smoking n=13,524 .

Table 2

Characteristics of women with known and unknown outcomes of pregnancy presenting in the first trimester (with no malaria or a single first trimester episode of malaria).

Characteristic	Unknown outcome N=2,376	Known outcome N=17,613	P-value
Age, yrs, mean ± SD, [range]	26 ±7 [13-46]	27±7 [13-52]	<0.001
Teenager < 20 y.o., n (%)	434/2,376 (18.3)	2605/17,612 (14.8)	<0.001
Gravidity, median (IQ range) [range]	2 (1-4) [1-16]	3 (2-5) [1-17]	<0.001
Parity, median (IQ range) [range]	1 (0-3) [0-13]	2 (1-3) [0-13]	<0.001
Primigravidae, n (%)	738/2,376 (31.1)	3,626/17,585 (20.6)	<0.001
Smokers, n (%)	619/2,270 (27.3)	4,320/14,390 (30.0)	0.008
Malaria 1st trimester, n (%)	363/2,376 (15.3)	945/17,613 (5.4)	<0.001
Signs of pregnancy loss at presentation, n (%)	219/2,370 (8.9)	1196/17,613 (6.8)	<0.001
Antimalarial Treatment			
Chloroquine	153/363 (42.1)	429/945 (45.4)	
Quinine	168/363 (46.3)	390/945 (41.3)	
Artesunate	36/363 (9.9)	92/945 (9.7)	0.025
Mefloquine	3/363 (0.3)	32/945 (3.4)	
Other	3/363 (0.8)	2/945 (0.2)	

SD-standard deviation; IQ range-inter-quartile range;
Missing data: Teenager n=1, primigravidae n=28, parity n=37, gravidity n=28, smoking n=3329, signs of pregnancy loss at first consultation n= 6.

Table 3.
Congenital abnormalities in the first trimester, single treatments.

Treatment	Age yrs	Gravidity (G) Parity (P)	EGA malaria treatment, wks	Abnormality	Critical period of organ formation	Proportion of abnormality in women treated with antimalarial	Proportion of same abnormality in women with no antimalarial treatment
Artesunate	33	G7 P4	12.5	Syndactyly foot - digits fused bilateral 2, 3 and 4 no toe nails	Toe nails week 14	2.3 (1/44)	2.5 (5/198)
Artesunate	21	G1 P0	3.9	Hydrocephalus	Brain 3-16 wks	2.3 (1/44)	3.0 (6/198)
Quinine	20	G2 P1	9.7	Cleft lip and palate	Palate- 6-8 wks	0.4 (1/258)	12.6 (25/198)
Chloroquine	25	G3 P2	13	Arthrogryposis	Limb rotation 8 wks	0.4 (1/260)	1.0 (2/198)

EGA – estimated gestational age.
